# Sacral Dimple, Conjunctiva, and Nipple as Less Obvious Pemphigus Vulgaris Locations around Natural Body Orifices: A Report of Three Cases

**DOI:** 10.3390/jcm11102855

**Published:** 2022-05-18

**Authors:** Magdalena Jałowska, Justyna Gornowicz-Porowska, Monika Bowszyc-Dmochowska, Marian Dmochowski

**Affiliations:** 1 Autoimmune Blistering Dermatoses Section, Department of Dermatology, Poznan University of Medical Sciences, 60-355 Poznan, Poland; mjalowska@ump.edu.pl; 2 Department and Division of Practical Cosmetology and Skin Diseases Prophylaxis, Poznan University of Medical Sciences, 60-806 Poznan, Poland; justynagornowicz1@poczta.onet.pl; 3 Cutaneous Histopathology and Immunopathology Section, Department of Dermatology, Poznan University of Medical Sciences, 60-355 Poznan, Poland; m.bowdmo@wp.pl

**Keywords:** pemphigus vulgaris, natural body orifices, sacral dimple, nipple, eye

## Abstract

In this paper, we present our own clinical-laboratory experience concerning three less obvious presentations of pemphigus vulgaris (PV) and discuss the pertinent literature. The involvement of the sacral dimple reported here for the first time, as well as the nipple and the eyes, could initially be misleading clinically. These less stereotypical localizations may occur due to the transition of different epithelia, each with varying levels of cadherin (desmoglein, desmocollin) and thus altered sensitivity to mechanical stress. The role of dermatologists who have experience in treating autoimmune blistering dermatoses is fundamental for identifying promptly the initial and exacerbating PV lesions in such unusual locations.

## 1. Introduction

Pemphigus is a group of rare, chronic, potentially life-threatening autoimmune bullous diseases of the skin and/or mucous membranes [1]. Pemphigus vulgaris (PV), pemphigus foliaceus, and paraneoplastic pemphigus (PNP) are the main subgroups of pemphigus. The most common subtype of pemphigus is PV, characterized by circulating IgG antibodies directed against desmoglein (DSG) 3 alone or both DSG 3 and DSG 1. Antibodies to DSGs cause loss of cell–cell adhesion between epithelial cells, which results in acantholysis and formation of intraepithelial vesicles/bullae (primary lesions) and erosions (secondary lesions) on the skin and/or mucous membranes. Pemphigus is diagnosed on the basis of clinical manifestations, direct immunofluorescence (DIF) examination of perilesional tissue, and serum testing for circulating autoantibodies against DSG 3 and DSG 1 using multiplex approaches based on ELISA or indirect immunofluorescence. The histological H + E staining of the lesional tissue, despite the fact that it is impossible to detect accompanying autoimmune phenomena, can reveal pemphigus-suggesting features, particularly if evaluated by an experienced pathologist.

The skin lesions in PV typically can be found on the scalp, face, trunk, axilla, groin, and pressure points [2]. Oral and mucosal lesions are seen in 80% to 90% of cases and may represent initial presentations of PV [2]. The frequency of otorhinolaryngologic (ENT) involvement has been described as pharynx (38–85%), larynx (40–85%), nasal cavity (11–76%), and ear (8–27%) [3]. Auricular findings in PV patients with otoendoscopic examination were confirmed and well described in 10.5% to 26.8% of patients [4,5]. Involvement of the female genital tract in pemphigus vulgaris is relatively rare. Vulvar lesions occur more commonly than vaginal lesions [6]. Literature data indicate the presence of nail lesions in up to 22–30% of PV patients [7]. PV lesions have a tendency to localize around natural body orifices. It is also suggested that skin appendages (hair follicles, eccrine glands) are affected by processes occurring in the course of pemphigus diseases [8]. It was speculated that PV tends to affect skin on those body parts where there is a transition between various kinds of epithelia: especially where the skin passes into the mucous membranes, in such places as the oral cavity, auricle, vestibule of the nose, anal orifice, clitoris, glans, urethra, nipple, and others [8]. DSG 3 expression predominates in these localizations, which may explain the more frequent localization of skin lesions in these areas. Sometimes, skin lesions in the course of pemphigus can assume very unobvious presentations. In our recent study, skin lesions in the navel area were present in 12 of 81 ethnic Poles with PV (14.8%) [9]. Only females (10 with mucocutaneous PV, 1 with mucosal dominant PV, and 1 with pemphigus vegetans) had lesions in the navel area. Therefore, we speculated that a causal relationship may exist between the female reproductive system and the pattern of expression of PV lesions around umbilicus. The possible underlying pathomechanism for the presence of PV lesions in the umbilicus includes expression of altered antigens linked with embryonic structures such as the umbilical cord that would stimulate IgG autoimmunity against DSG isoforms [10].

The initial manifestation of PV at a clinically non-typical body site is a diagnostic challenge for a dermatologist. In some cases, awareness that PV can infrequently affect locations around natural body orifices should help to establish a PV diagnosis. For patients already diagnosed with PV, skin lesions in an “unusual” body site always require a detailed differential diagnosis.

In this paper, we present three PV patients showing sacral dimple, ocular, and nipple involvement and discuss the pertinent literature concerning these natural body orifices in pemphigus.

## 2. Case Report: Sacral Dimple Involvement

**Figure 1:**
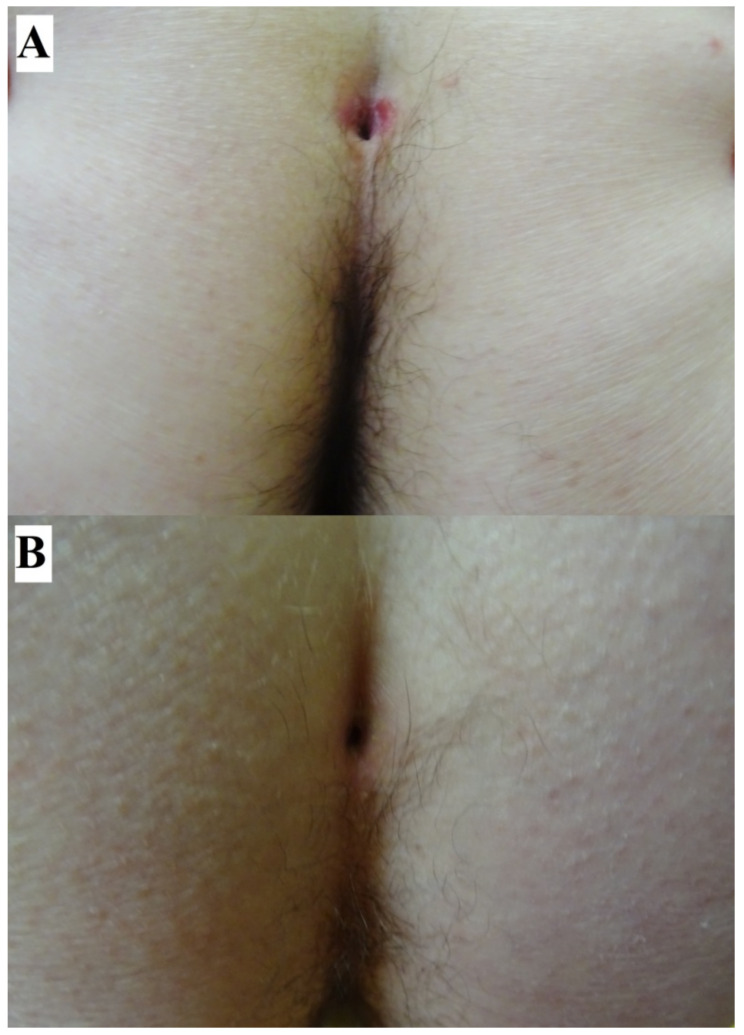
A 48-year-old woman with PV affecting the sacral dimple (**A**). Healing of lesions around the sacral dimple after implementing treatment for pemphigus (**B**).

A 48-year-old woman (Figure 1) with a 2-month history of intermittent painful oral blisters on buccal mucosa, lips, and throat as well as skin lesions also affecting the sacral dimple was admitted to our outpatient clinic. A DIF of perilesional skin showed pemphigus IgG4 (+++) and C3 (++) deposits. A multi-analyte ELISA revealed elevated levels of IgG serum anti-DSG 3 (6.57) antibodies (cut-off value: 1.0). Both findings confirmed a PV diagnosis (Figure 1). A month before that, the patient had been hospitalized in the otolaryngology department due to lesions located on the mucous membranes of the mouth and throat. A histopathological examination of the palate mucosa was performed, but the reading only revealed a chronic deep inflammation without any suggestions of its cause. The patient was treated in the department of dermatology. Methylprednisolone was administered intravenously (1000 mg per dose, for 3 days) with clinical improvement. Subsequently, oral methylprednisolone treatment was implemented (an initial dose of 32 mg/per day for 2 weeks, then tapered at 2-week intervals), and a durable clinical remission was obtained. During hospitalization in the department of dermatology, a chest X-ray and an abdominal ultrasound were performed. Detailed laboratory tests including tumor markers were performed. There were no abnormalities. The patient did not have any comorbidities.

Sacral dimples (sacrococcygeal or orcoccygeal dimples or pits) are the most common cutaneous anomaly found during neonatal spinal examinations [11]. They are defined as deep or shallow depressions occurring at the lower sacral region close to or within the natal cleft. Isolated sacral dimples are innocuous [11]. The majority of these tracts terminate in epidermoid or dermoid cys [12]. The most widely accepted theory regarding the embryogenesis of congenital dermal sinus tracts proposes that they arise through faulty disjunction of the neuroectoderm from the overlying cutaneous ectoderm between the third and eighth weeks of gestation [12]. Cadherin-mediated cell–cell junctions form early in embryonic development [13]. Adherens junctions are required for preimplantation development, blastocoel cavity formation, and/or epithelial polarization.

An expression of altered antigens linked with embryonic structures such as lining of the spinal cord (thecal sac) that would stimulate IgG autoimmunity against DSG isoforms is a plausible explanation for the presence of pemphigus lesions located in the area of the sacral dimple. Moderate expression of radially arranged DSG was present around the central canal of the spinal cord throughout all studied developmental stages [14]. Mechanical stress due to friction from tight clothes could be yet another reason for the appearance of skin lesions around the sacral dimple.

Skin lesions around the sacral dimple are very rare but always require detailed diagnostics to rule out pilonidal cysts, psoriasis, eczema, and hidden malignancy. Such lesions should be diagnosed subject to careful clinical examination and if necessary histopathological evaluation. In some cases, a direct immunofluorescence (DIF) examination is a crucial diagnostic procedure. For pemphigus, a diagnosis may be facilitated by presence of Nikolsky’s sign and a dermoscopy examination.

## 3. Case Report: Ocular Involvement

**Figure 2:**
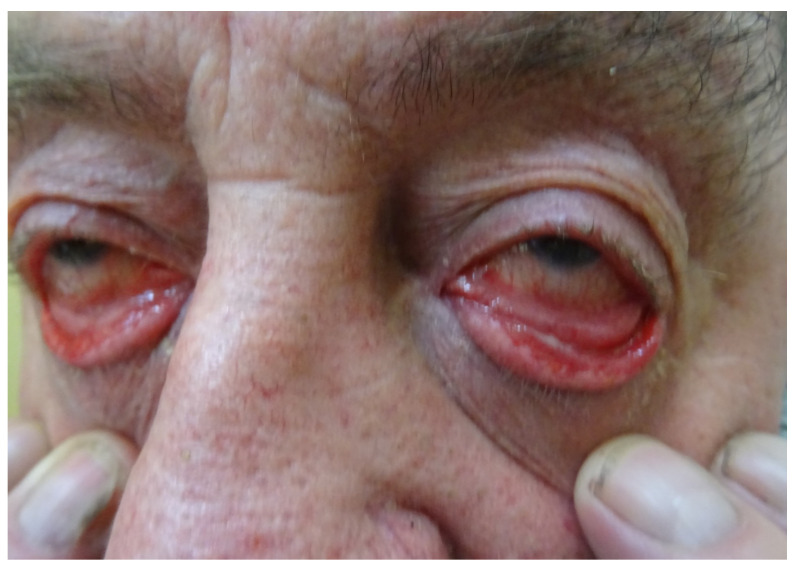
A 73-year-old man with an exacerbation of PV bilaterally involving mainly conjunctiva.

A 73-year-old man with a three-month history of an apparent PV exacerbation involving mainly conjunctiva was seen in our outpatient clinic. An ophthalmological examination revealed ectropion of the lower eyelid and inflammatory changes in the eyelid conjunctiva. Minor erosions of the cornea of the right and left eyes were found. During this bilateral ocular exacerbation (Figure 2), taking place while the patient was on a daily oral maintenance methylprednisolone dose of 4 mg, a multi-analyte ELISA revealed increases of the levels of IgG serum anti-DSG 1 (3.68) and anti-DSG 3 (5.51) antibodies compared to the follow-up values, indicating the relapsing stage of PV. The patient had contracted the mucocutaneous disease 2 years beforehand. At the beginning of the disease, lesions were located in the nasal cavity and in the mouth. A histopathological examination of the nasal cavity mucosa was performed by the otolaryngology department. Unfortunately, the sample was non-diagnostic. Initially, a DIF of perilesional neck skin performed in the department of dermatology showed pemphigus IgG4 (+++) and C3 (+/−) deposits, whereas a multi-analyte ELISA revealed elevated levels of IgG serum anti-DSG 1 (2.00) anti-DSG 3 (7.37) antibodies. Both these findings eventually confirmed a diagnosis of PV. Therefore, the patient was treated with oral doxycycline 200 mg daily for 4 weeks and with oral methylprednisolone (an initial dose of 32 mg/per day for 3 weeks, then tapered at 2-week intervals). During the follow-up, after one year of treatment with tapering doses of oral methylprednisolone, the levels of IgG serum anti-DSG1 antibodies were below the cut-off value (0.21), and we observed a lowering of the level of IgG serum anti-DSG 3 (3.56) antibodies. All ELISA cut-off values were 1.0. Because of the ocular relapse, the dose of oral methylprednisolone was increased to 32 mg daily, and oral dapsone was started (an initial dose of 50 mg daily). Ophthalmologically, the patient was treated with ofloxacin eye drops and gel containing dexpanthenol with polyacrylic acid. This combined treatment markedly improved the ocular condition. The patient has been suffering from chronic arterial hypertension and chronic ischemic heart disease. The patient also underwent thyroidectomy for nodular goiter of the thyroid gland.

Expression of the majority of desmosomal molecules in human conjunctiva is similar to the expression of DSGs in the middle of the epidermis [15]. DSG 3 is expressed on the entire ocular surface, including the cornea, bulbar, and palpebral conjunctiva [16]. Expressions of DSG 1 and DSG 3 are uniform in the cornea and sclera. DSG 3 is also expressed in deeper structures of the eye, like the retina [16].

Incubation of human specimens with PV-IgG for 12 h caused blistering in the suprabasal layers of the conjunctiva and a reduction of DSG 1 and DSG 3 protein levels [15]. Only a slight depletion of DSG 3 was detectable. DSG 1 depletion was readily evident by immunoblotting, indicating a participation of DSG 1 autoantibodies in conjunctival blistering. PV cutaneous lesions adjacent to the medial angle of the eye are usually more prominent compared to the lateral one. Typically, PV blistering in the conjunctiva occurs in the suprabasal layers which matches the findings in the epidermis [17].

The eyes are less frequently involved than oral mucosa [2], and ocular findings are seen in between 7% and 16.5% of PV patients [16,17,18]. Most commonly, ocular presentations in PV are unilateral [16]. This may be due to antigen mosaicism concentration or pathergy [16]. Studies have shown non-cicatricial conjunctivitis and blepharitis to be the most common pemphigus ocular presentations. Erosions on the eyelids and the conjunctiva are only rarely observed [19]. Case reports range from only mild conjunctivitis to severe types with cicatrization, corneal ulceration, subconjunctival scarring, symblepharon, trichiasis, entropion, corneal opacities, ankyloblepharon, and corneal perforation [20,21]. Ocular involvement has been reported as a signal of severe disease or relapse and tends to occur several months after the onset of skin or other mucosal lesions [19]. On the other hand, there is evidence to suggest that ocular symptoms can precede the onset of mucus membrane and skin lesions, suggesting that ocular involvement may be underdiagnosed in patients [17,19].

Recalcitrant conjunctivitis with conjunctival blisters should warrant a workup for PV [16]. There is evidence to suggest that ocular symptoms can precede the onset of mucous membrane and skin lesions, suggesting that ocular involvement may be underdiagnosed in PV patients [18,20]. Ocular PV needs to be differentiated from ocular mucous membrane pemphigoid (MMP, formerly known as cicatricial pemphigoid). Progressive scarring conjunctivitis is a clinical feature of MMP, whereas scarring is less common in PV. PV should also be differentiated from PNP, particularly in patients exhibiting stubborn conjunctival or anal lesions. PNP is associated with neoplasms, which can be benign or malignant. The neoplasm is usually chronic lymphocytic leukemia, lymphoma (non-Hodgkin’s type), Castleman’s disease, or thymoma [2]. Still, there are cases of malignancy-associated PV without laboratory features of PNP. A multiplex ELISA containing envoplakin combined with a clinical examination and a DIF should be helpful for routine laboratory diagnostics and differentiation of PNP [22].

## 4. Case Report: Nipple Involvement

**Figure 3:**
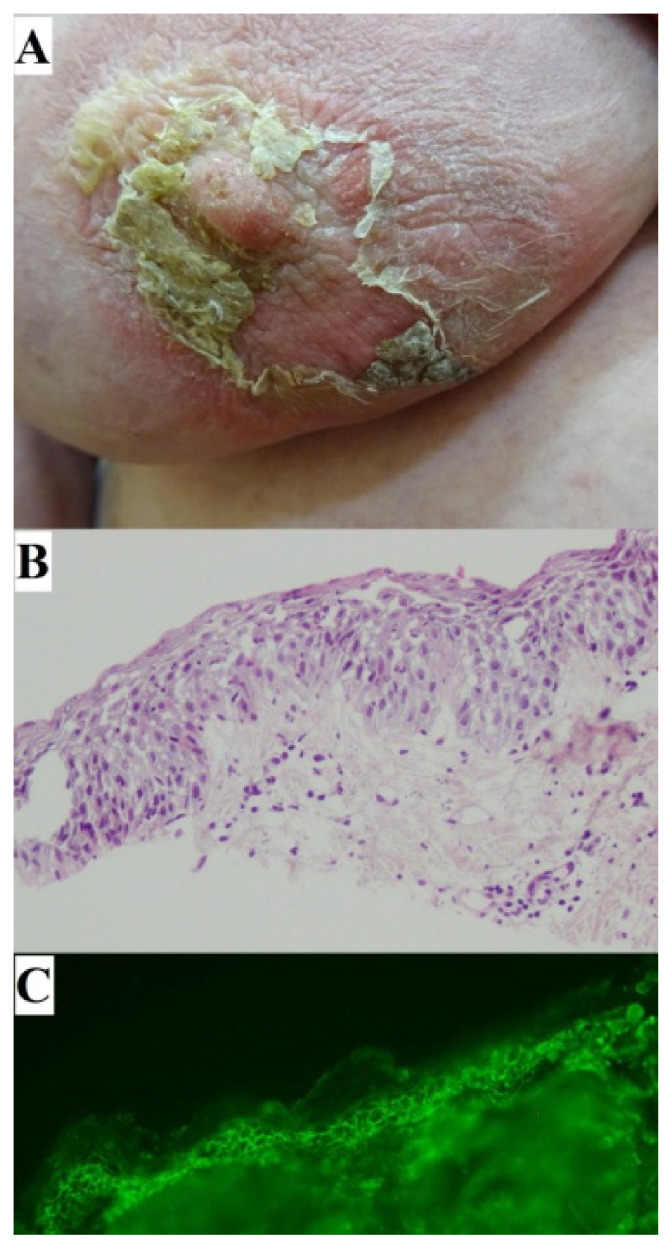
A 63-year-old woman with PV lesions on the right breast around the nipple with impetiginization (**A**). Acantholytic keratinocytes in the upper epidermis in H + E histology (original objective magnification ×20) (**B**). IgG4 (++) pemphigus deposits revealed with a DIF visualized using a short arc mercury lamp-operated microscope (original objective magnification ×20) (**C**).

A 63-year-old woman was seen in our outpatient clinic because of a two-month history of widespread painful mucocutaneous lesions involving the right breast around the nipple with impetiginization, the lower abdomen, the area around navel, eyelids, and buccal mucosae. A DIF of perilesional skin showed pemphigus IgG (+), IgG1 (+), IgG4 (++), and C3 (+) deposits. A multi-analyte ELISA revealed elevated levels of IgG serum anti-DSG 1 (7.91) and anti-DSG 3 (1.73) antibodies (cut-off for both: 1.0). Both these findings confirmed a PV diagnosis (Figure 3). A H + E histology examination demonstrated acantholytic keratinocytes in the upper epidermis. The patient was treated in the department of dermatology. Methylprednisolone was administered intravenously (2.5 g in total for 3 days). Subsequently, a combination therapy comprising oral methylprednisolone (an initial dose of 32 mg/per day for 2 weeks, then tapered at 2-week intervals) and oral cyclophosphamide (150 mg per day) was introduced. Clinical remission was obtained. Cyclophosphamide had to be discontinued due to hemorrhagic cystitis. Rosuvastatin was also discontinued due to worries over sustaining an autoimmune blistering condition due to this compound. The patient has been suffering from chronic arterial hypertension and type 2 diabetes. The patient also underwent thyroidectomy for nodular goiter of the thyroid gland.

The nipple is covered by pigmented squamous epithelium. Moreover, keratin-producing squamous cells of the epidermis extend into major ducts for 1–2 mm, and there is an abrupt transition from squamous cells to normal luminal/myoepithelial lining of ducts. The epithelium of exosecreting ducts extends to directly reach the skin of the nipple [23]. DSG 3 should be expressed there in the same manner as in the squamous epithelium elsewhere, which may explain the tendency of PV lesions to localize around the nipple [23,24].

In the literature, we found a description of PV and breast cancer coexisting in the area of the nipple. A 54-year-old woman had a 3-month history of a rash localized on her right breast and nipple. Six months before this presentation, she was diagnosed with PV. The skin overlying her right breast was covered with multiple confluent erosions, hyperkeratotic scales, and crusts. A DIF of the perilesional skin showed IgG and C3 pemphigus deposits, whereas an ELISA revealed high levels of IgG antibodies to DSG 1 and 3. A computed tomography scan and a subsequent breast biopsy confirmed the presence of an invasive triple negative ductal carcinoma [25]. Garcia-Souto described a patient with eroded plaques involving the breasts [26]. The patient did report wearing a tight bra frequently which could cause mechanical stress pemphigus. Guyton et al., examined a development of the ambiguous skin lesion involving the areola of a 37-year-old woman [27]. Clinically the lesion was compatible with Paget’s disease; however, a histological evaluation identified features of PV [27]. The underlying role of plasminogen activator in the molecular pathology of both diseases was discussed [27]. In this respect, malignant keratinocytes exhibit altered DSG 2 and DSG 3 expression patterns, the proteins targeted by pemphigus autoimmunity [28].

For a differential diagnosis of PV located on the nipple, it is necessary to take into account other diseases. Nipple hyperkeratosis was associated with malignancies such as cutaneous lymphoma and malignant acanthosis nigricans [29]. Nipple eczema also has to be taken into consideration during a differential diagnosis. This can be caused by irritant or allergic contact dermatitis or even be a feature of atopic dermatitis. It presents as pruritic erythematous plaques on the nipple and may feature crusts, scales, and fissuring mammary Paget’s disease. Those disease entities must be also included in a differential diagnosis. Clinicians should be aware of the possibility of underlying malignancies in pemphigus patients experiencing localized flares [29]. A dermoscopy examination can be very helpful for a differential diagnosis. In our patient, Nikolsky’s sign made the diagnosis easier at the clinical level.

## 5. Discussion of the Issues Common to the Described PV Locations

In this paper, we present three less obvious presentations of PV. The triggering factors of PV still remain unclear. These multifactorial triggers involve interactions between environmental influences and genetic predispositions [30]. The strong association with MHC class II molecules indicates a T cell-mediated role, and blister tissue shows elevated IL-17 and IL-23 [31]. According to the current understanding of PV etiopathology, DSG 3 is the principal antigen targeted by a predominantly IgG4 subclass of autoreactive antibodies [32].

We speculate that the transition between various kinds of epithelia lining natural body orifices may be accompanied by peculiarities of expression patterns of isoforms of both DSG and desmocollin, and therefore, it may facilitate the location of PV lesions in such sites of privilege. Conceivably, the skin immune system may act more vigorously at external–internal interfaces combating noxious environmental factors compared to other cutaneous areas, which may initiate the development of an autoimmune response characterizing PV in genetically prone individuals.

Four DSG isoforms (DSG 1–4) have been identified in humans. These isoforms are differentially expressed in various epithelial tissues. DSG 2 and DSG 3 are mainly distributed throughout the lower layers of the epidermis, whereas DSG 1 is expressed at higher levels in the upper layers, with low to undetectable levels in the basal layers. DSG 3 has a strong basal distribution associated with proliferating cells [33]. DSG 4 is primarily expressed in the hair follicle and in the granular layer [34]. One explanation for the variable distribution of DSG isoforms in different epithelia might be the isoforms providing adhesion that is appropriate for the specific types of stress to which these epithelia are subjected [35]. On the other hand, the differential distribution of DSG isoforms might influence the differentiation and/or function of these epithelia [33]. Expressing DSG 3 in the upper layers of the epidermis using an involucrin promoter resulted in resemblance to oral mucosa, reduction in epidermal barrier function, and early postnatal lethality because of extensive water loss [35]. Cells with low levels of DSG 3 expression (DSG 3-dim) exhibited increased colony forming efficiency and enhanced skin regeneration capability relative to cells with high levels of DSG 3 expression (DSG 3-bright) [36,37]. DSG 3-expressing tissues, e.g., skin and oral mucosa, are exposed daily to various stresses, such as mechanical stretching, that could induce p53 [38]. Concerning the issue of the tissue stress, DSG 3 [38] was experimentally shown to be a stress-protective protein in keratinocytes via negative regulation of p53. Therefore, loss of function of DSG 3 caused by PV autoimmunity may decrease the tolerance of keratinocytes to external stress stimuli operating at natural body orifices.

Mechanical stress could have triggered blisters in our patients at clinically non-typical body sites. Applying tangential pressure by a thumb to normal skin in a patient with pemphigus results in a force that dislodges the upper layers of the epidermis from the lower layers (Nikolsky’s sign). An additional explanation for such localization of skin lesions in pemphigus, apart from variable DSG expression, is mechanical trauma. This mechanism is feasible for our PV patients exhibiting lesions localized in the area of the nipple, the sacral dimple, and the conjunctivae. Most commonly ocular presentations of PV are unilateral. This may be due to pathergy in which in which the patient rubs one eye and induces lesions formations [16]. Trauma-induced pemphigus has been reported mainly after surgery, radiation, or burns [39]. Rashid et al., described a patient with blisters which initially appeared around a surgical incision, 2 weeks following an appendectomy [30]. Mehregan et al., also reported a case of pemphigus arising in areas of surgical manipulation after rhinoplasty and hair transplantation occurring 3 months and 1 year after surgery, respectively [40]. Duick et al., presented a case of pemphigus that started in a Mohs surgical wound after an excision of a squamous cell carcinoma (SCC) from a 49-year-old woman [41]. Jetter et al., described pemphigus vegetans after cryosurgery for actinic keratosis (AKs) at the temple and forehead [42]. A retrospective study of trauma-induced pemphigus found major surgery procedures (abdominal, chest, and orthopedic) as the most frequent trigger. In addition, minor procedures such as periodontal procedures, electrosurgery, or laser surgery could also trigger pemphigus [43]. Out of 36 cases of surgically induced PV, thirteen were in patients without pre-existing pemphigus [44]. The pathogenesis of mechanical trauma in PV is unknown and is probably multifactorial [44]. It could be mediated by proinflammatory mediators released in a nonspecific manner following injuries, through non-antigen-specific mechanisms by modulating C3, plasminogen activator, and plasminogen activator inhibitor expression [45]. These cytokines may act as facilitators in an already pemphigus-prone skin [45]. Furthermore, surgical trauma may trigger an epitope-spreading phenomenon [42,44]. Nevertheless, another explanation might be that epidermal injury may expose DSG 1 and 3 and lead to new autoantibody formation in genetically susceptible patients or to activation of pre-existing antibodies already present in low (subclinical) titers [42,44]. An immunocompromised district (ICD), where edema and nerve injury paved the way for a buildup of immunoglobulins and immune cells, is probably associated with the development of pemphigus at postoperative sites [46]. Moreover, there are several hypotheses concerning induction of localized ABDs in regions of prior trauma (i.e., scars) [47,48,49], including disruption of normal keratinocyte differentiation and local changes in vascular and connective tissue function. It might result in increased susceptibility of keratinocytes to circulating pemphigus autoantibodies via defective or deficient levels of cadherins [46].

Finally, we suspect that the stubborn inflamed PV lesions around natural body orifices described here are the result of focal autoimmune response maintained by diffuse ectopic lymphoid-like structures (ELSs) [50], which may also be formed at such PV sites of privilege. Those diffuse ELSs are transient ectopic lymphoid aggregates responsible for the production of autoantibodies to DSG 1 and 3 mediating the active phase of pemphigus. Because of the presence of the ELSs, local B cells may not be easily inhibited by systemic therapy, including rituximab, leading to treatment-resistant pemphigus lesions. Pemphigus lesions might act as a niche, supporting in situ B cell differentiation and clonal expansion [50]. Therefore, we are tempted to speculate that isolated stubborn lesions seen in PV patients, despite systemic anti-pemphigus therapies, wherever technically possible, should be surgically excised. Concomitant systemic anti-pemphigus therapies should prevent the formation of any new PV lesions potentially induced by trauma caused by surgical excision.

## 6. Conclusions

Blisters and their evolutionary lesions can be present in unobvious anatomic areas that are not always inspected during routine clinical examinations. Therefore, the role of the dermatologist is fundamental in taking into account PV, and in the evaluation of the real disease extension. Stubborn lesions around natural body orifices might be a valuable clinical hint for carrying out immunopathological testing to unequivocally diagnose incipient or exacerbated PV.

## Data Availability

All results can be found in the patients’ medical records.

## References

[1] Didona D., Maglie R., Eming R., Hertl M. (2019). Pemphigus: Current and future therapeutic strategies. Front. Immunol..

[2] Feizi S., Roshandel D. (2019). Ocular manifestations and management of autoimmune bullous diseases. J. Ophthalmic Vis. Res..

[3] Ohki M., Kikuchi S. (2017). Nasal, oral, and pharyngolaryngeal manifestations of pemphigus vulgaris: Endoscopic ororhinolaryngologic examination. Ear Nose Throat J..

[4] Kavala M., Altıntaş S., Kocatürk E., Zindancı İ., Can B., Ruhi Ç., Turkoglu Z. (2011). Ear, nose and throat involvement in patients with pemphigus vulgaris: Correlation with severity, phenotype and disease activity. J. Eur. Acad. Dermatol. Venereol..

[5] Robati R.M., Rahmati-Roodsari M., Dabir-Moghaddam P. (2012). Mucosal manifestations of pemphigus vulgaris in ear, nose, and throat; before and after treatment. J. Am. Acad. Dermatol..

[6] Malik M., Razzaque A. (2005). Involvement of the female genital tract in pemphigus vulgaris. Obstet. Gynecol..

[7] Pietkiewicz P., Bowszyc-Dmochowska M., Gornowicz-Porowska J., Dmochowski M. (2018). Involvement of nail apparatus in pemphigus vulgaris in ethnic Poles is infrequent. Front. Med..

[8] Dmochowski M. (2007). Natural body orifices and mucous membranes in pemphigus vulgaris. Dermatol. Klin..

[9] Jałowska M., Gornowicz-Porowska J., Seraszek-Jaros A., Bowszyc-Dmochowska M., Kaczmarek E., Dmochowski M. (2022). Clinical significance of umbilical region involvement in pemphigus vulgaris in a series of 81 ethnic Poles: A comparative analysis of the distribution of lesions in two infrequent locations. Adv. Dermatol. Allergol. Post. Derm. Alerg..

[10] Aoki W., Maruta C., Santi C. (2010). Umbilical involvement in pemphigus vulgaris. J. Am. Acad. Dermatol..

[11] Lee A.C., Kwong N.S. (2007). Management of sacral dimples detected on routine newborn examination: A case series and review. HK J. Paediatr..

[12] Mostafa M., Nasef N., Barakat T., El-Hawary A.K., Abdel-Hady H. (2013). Acute flaccid paralysis in a patient with sacral dimple. World J. Clin. Pediatr..

[13] Gloushankova N.A., Rubtsova S.N., Zhitnyak I.Y. (2017). Cadherin-mediated cell-cell interactions in normal and cancer cells. Tissue Barriers.

[14] Saraga-Babi M., Stefanovi V., Saraga M., Wartiovaara J., Lehtonen E. (2002). Expression of intermediate filaments and desmosomal proteins during differentiation of the human spinal cord. Acta Histochemica.

[15] Vielmuth F., Rötzer V., Hartlieb E., Hirneiß C., Waschke J., Spindler V. (2016). Pemphigus autoantibodies induce blistering in human conjunctiva. Invest. Ophthalmol. Vis. Sci..

[16] Memar O., Jabbehdari S., Caughlin B., Djalilian A.R. (2020). Ocular surface involvement in pemphigus vulgaris: An interdisciplinary review. Ocul. Surf..

[17] Daoud Y.J., Cervantes R., Foster C.S., Ahmed A.R. (2005). Ocular pemphigus. J. Am. Acad. Dermatol..

[18] Akhyani M., Keshtkar-Jafari A., Chams-Davatchi C., Lajevardi V., Beigi S., Aghazadeh N., Rayati Damavandi M., Arami S. (2014). Ocular involvement in pemphigus vulgaris. J. Dermatol..

[19] Gianniou C., Dirani A., Ferrini W., Marchionno L., Decugis D., Deli A., Ambresin A., Mantel I. (2014). Cicatricial changes in ocular pemphigus. Eye.

[20] Baykal H.E., Pleyer U., Sonnichsen K., Thiel H.J., Zierhut M. (1995). Severe eye involvement in pemphigus vulgaris. Ophthalmologe.

[21] Alkatan H., Alzahem T., Srivastava S. (2018). Histopathology of the Ocular Surface. Histopathology: An Update.

[22] Gornowicz-Porowska J., Seraszek-Jaros A., Bowszyc-Dmochowska M., Bartkiewicz P., Kaczmarek E., Dmochowski M. (2018). Clinical evaluation of a multiparametric ELISA as a rapid tool for routinely diagnosing IgG-mediated autoimmune blistering dermatoses in ethnic Slavs. J. Clin. Lab. Anal..

[23] Stolnicu S., Stolnicu S., Alvarado-Cabrero I. (2018). Histology of the Normal Breast, Normal Changes, and Abnormalities of Breast Development. Practical Atlas of Breast Pathology.

[24] Cieśla S., Wichtowski M., Poźniak-Balicka R., Murawa D. (2020). The surgical anatomy of the mammary gland (part 1.) General structure, embryogenesis, histology, the nipple-areolar complex, the fascia of the glandular tissue and the chest wall. J. Oncol..

[25] Maglie R., Montefusco F., Senatore S., Massimiliano D’Erme A., Bagnoni G., Antiga E. (2020). Localized pemphigus exacerbation associated with underlying breast cancer. JAAD Case Rep..

[26] Garcia-Souto F. (2020). Eroded plaques involving the breasts: A unique location of pemphigus vulgaris. An. Bras. Dermatol..

[27] Guyton D.P., Stakleff K.S., Regula E. (2003). Pemphigus vulgaris mimicking Paget’s disease of the breast. Breast J..

[28] Pietkiewicz P., Gornowicz-Porowska J., Bowszyc-Dmochowska M., Jagielska J., Helak-Łapaj C., Kaczmarek E., Dmochowski M. (2014). Discordant expression of desmoglein 2 and 3 at the mRNA and protein levels in nodular and superficial basal cell carcinoma revealed by immunohistochemistry and fluorescent in situ hybridization. Clin. Exp. Dermatol..

[29] Çağlar B., Saral C., Akay B.N., Kundakçı N. (2019). Paraneoplastic pemphigus and nipple hyperkeratosis triggered with Castleman disease. Turk. J. Dermatol..

[30] Rashid A., Wang J., Fu P., Wang W., Xie H. (2015). Pemphigus vulgaris associated with surgery: A rare association. Indian J. Dermatol. Venereol. Leprol..

[31] Xue J., Su W., Chen Z., Ke Y., Du X., Zhou Q. (2014). Overexpression of interleukin-23 and interleukin-17 in the lesion of pemphigus vulgaris: A preliminary study. Mediat. Inflamm..

[32] Nagel A., Lang A., Engel D., Podstawa E., Hunzelmann N., de Pita O., Borradori L., Uter W., Hertl M. (2010). Clinical activity of pemphigus vulgaris relates to IgE autoantibodies against desmoglein 3. Clin. Immunol..

[33] Teh M.T., Fortun F., Kenneth E., Wan H. (2011). A molecular study of desmosomes identifies a desmoglein isoform switch in head and neck squamous cell carcinoma. J. Oral Pathol. Med..

[34] Delva E., Tucker D.K., Kowalczyk A.P. (2009). The Desmosome. Cold Spring Harb. Perspect. Biol..

[35] Elias P.M., Matsuyoshi N., Wu H., Lin C., Wang Z.H., Brown B.E., Stanley J.R. (2001). Desmoglein isoform distribution affects stratum corneum structure and function. J. Cell Biol..

[36] Wan H., Yuan M., Simpson C., Allen K., Gavins F.N., Ikram M.S., Basu S., Baksh N., O’Toole E.A., Hart I.R. (2007). Stem/progenitor cell-like properties of desmoglein 3dim cells in primary and immortalized keratinocyte lines. Stem Cells.

[37] Koster M.I., Kim S., Roop D.R. (2005). P63 deficiency: A failure of lineage commitment or stem cell maintenance?. J. Investig. Dermatol. Symp. Proc..

[38] Rehman A., Cai Y., Hünefeld C., Jedličková H., Huang Y., Teck Teh M., Sharif Ahmad U., Uttagomol J., Wang Y., Kang A. (2019). The desmosomal cadherin desmoglein-3 acts as a keratinocyte anti-stress protein via suppression of p53. Cell Death Dis..

[39] Jang H.W., Chun S.H., Lee J.M., Jeon J., Hashimoto T., Kim I.H. (2014). Radiotherapy-induced pemphigus vulgaris. J. Dermatol..

[40] Mehregan D.R., Roenigk R.K., Gibson L.E. (1992). Postsurgical pemphigus. Arch. Dermatol..

[41] Duick M.G., Zaks B., Moy L.R., Kaplan R.P. (2001). Mohs micrographic surgery-induced pemphigus. Dermatol. Surg..

[42] Jetter N., Cerci F.B., Pandher K., Krunic A.L. (2021). Pemphigus vegetans developing after Mohs micrographic surgery and cryotherapy. An. Bras. Dermatol..

[43] Sagi L., Trau H. (2011). The Koebner phenomenon. Clin. Dermatol..

[44] Daneshpazhooh M., Fatehnejad M., Rahbar Z., Balighi K., Ghandi N., Ghiasi M. (2016). Trauma-induced pemphigus: A case series of 36 patients. J. Dtsch. Dermatol. Ges..

[45] Vinay K., Kanwar A.J., Saikia U.N. (2013). Pemphigus occurring at tuberculin injection site: Role of cytokines in acantholysis. Indian J. Dermatol. Venereol. Leprol..

[46] Patel P.M., Gibson F.T., Amber K.T. (2020). Recurrent pemphigus foliaceus at the site of a surgical scar following total knee arthroplasty. J. Clin. Aesthet. Dermatol..

[47] Milani-Nejad N., Chung C., Kaffenberger J. (2019). Recurrence of localized pemphigus foliaceus induced by knee replacement. J. Clin. Aesthet. Dermatol..

[48] Tolkachjov S.N., Frith M., Cooper L.D., Harmon C.B. (2018). Pemphigus foliaceus demonstrating pathergy after Mohs micrographic surgery. Dermatol. Surg..

[49] Rotunda A.M., Bhupathy A.R., Dye R., Soriano T.T. (2005). Pemphigus foliaceus masquerading as postoperative wound infection: Report of a case and review of the Koebner and related phenomenon following surgical procedures. Dermatol. Surg..

[50] Zhou S., Liu Z., Yuan H., Zhao X., Zou Y., Zheng J., Pan M. (2020). Autoreactive B cell differentiation in diffuse ectopic lymphoid-like structures of inflamed pemphigus lesions. J. Investig. Dermatol..

